# Trust in close relationships revisited

**DOI:** 10.1177/02654075251346105

**Published:** 2025-05-27

**Authors:** Omar J. Camanto, Lorne Campbell

**Affiliations:** 6221 University of Western Ontario, Canada

**Keywords:** Trust, romantic relationships, measurement, invariance testing, bifactor modeling

## Abstract

Trust is widely regarded as a fundamental psychological concept in the study of relationships yet—rather than being investigated as a primary, material focus—is often relegated to serving as a proxy for and/or a means to facilitate the exploration of aspects of relationship quality and functioning. We conducted two measurement-focused studies to garner deeper insight into the nature of trust in romantic relationships and explore an account of trust that reframes its development as a process of construction, rather than addition. In Study 1 (*N* = 494), we explored the nature and structure of the construct of trust as put forward by Rempel et al. (1985). In Study 2 (*N* = 847), we then confirmed our findings from Study 1 and, using measurement invariance techniques and a refined version of Rempel et al.'s (1985) assessment, investigated the extent to which the trust of individuals involved in newly-formed relationships (*n*_Newly-formed_ = 387) versus individuals involved in long term relationships (*n*_Long-term_ = 460) reflect different conceptualizations of the construct. Across both studies, we (a) identified a compilation of items reflecting key factors of trust—Predictability (3 items), Dependability (4 items), and Faith (10 items)—resembling Rempel et al.'s (1985) framework; and (b) found broad sameness in the construct of trust across newly-formed and long-term relationships but also some granular differences that potentially suggest how the construct may change across stages of relationship development.

## Introduction

The concept of trust is a key feature of theories and models focusing on functioning and well-being in romantic relationships (Reis, 2007, 2012; Simpson, 2007). The work of Rempel et al. (1985) is a landmark contribution that provided a theoretically rich foundation for capturing the trust people place in their intimate partners. Their theoretically derived measure and model for the development of trust is the primary source for modern thinking and research on the topic. However, research has not yet systematically evaluated the proposed factor structure of Rempel et al.’s (1985) trust scale, nor the similarities and differences in the nature of trust at different relationship stages. Additionally, the little research that has assessed the development of trust presents a potential paradox: people in new relationships report placing similarly high levels of trust in their romantic partners compared to those in long-lasting relationships (e.g., Fletcher et al., 2000; Larzelere & Huston, 1980).

With the present research, we conducted two measurement-focused studies to attain deeper insight into the nature of trust and explore an account of trust that reframes its development as a process of construction, rather than addition. In Study 1, we evaluated the soundness of Rempel et al.’s (1985) trust scale with a large sample of romantically-attached individuals to first establish how to best model trust’s elements. In Study 2, we then used bifactor modeling and invariance testing techniques with large, independent samples of individuals involved in relationships at different stages to capture divergences in the conceptualization of trust as a function of relationship maturation.

### Rempel et al.’s (1985) Instantiation of trust

#### The concept of trust

Trust can stem from evidence—data collected from past experiences—and/or faith— conviction taken as truth without any supporting evidence. In romantic relationships, the components of trust that concern evidence include perceptions of the partner’s predictability and dependability. Predictability refers to the perceived likelihood of successfully foretelling the future actions of the partner. Perceptions concerning a partner’s predictability are thought to be based on observations of the nature and consistency of the partner’s behaviours (Holmes & Rempel, 1989). Dependability refers to the degree to which the partner is perceived to embody a set of qualities approximating a “dependable archetype” (Holmes & Rempel, 1989, p. 191). Specifically, people would be more likely to regard their partner as dependable when their partner has solidified themselves as, for example, honest, cooperative, and benevolent. Although dependability, much like predictability, bears on the behavioural outputs of the partner, dependability is slightly more nuanced in that it involves the abstraction of underlying narratives and trait attributions from the partner’s outputted behaviours (Holmes & Rempel, 1989; Simpson, 2007).

Unlike predictability and dependability, faith captures a sense of conviction untethered to evidence, enabling one to dismiss doubts concerning the unknown future (Holmes & Rempel, 1989; Murray & Holmes, 2010). With faith in their partner and the future of the relationship, people can transcend the need for supportive evidence and act *as if* the future will unfold as desired (Boon & Holmes, 1991).

#### The measurement of trust

To establish their measure, Rempel et al. (1985) first created a compilation of 26 items presumed to reflect trust in a partner across the theorized dimensions and administered them to a sample of 47 long-term couples. The authors conducted an exploratory factor analysis (EFA) to analyze item responses and removed items on the grounds of (1) low item-total correlations and (2) poor loadings. The resulting 17-item questionnaire comprised a 5-item subscale reflecting predictability, a 5-item subscale reflecting dependability, and a 7-item subscale reflecting faith.

The authors regarded their research as an initial step towards gaining deeper insight into the nature of the theoretical entity of trust. However, the soundness and validity of their framework for capturing trust has undergone little psychometric investigation as most research emanating from their perspective has prioritized calculating an overall score to correlate with other measures of relationship functioning.

### The development of trust

The concept of trust in romantic relationships has been approached from at least two major theoretical perspectives: attachment theory and interdependence theory (Simpson, 2007). According to attachment theory, trust is one component of individual differences in attachment orientations that begin developing in childhood. Early interactions with core attachment figures during infancy are thought to later be crystalized into lasting mental representations of “self” and “others” (Hazan & Shaver, 1987; Mikulincer & Shaver, 2007). The responsiveness of the attachment figures to the infant’s needs is thought to shape the infant’s mental representations of “self” and/or “others”, leading to the formation of either a more secure or more insecure attachment orientation. Such attachment (in)security furnishes intuitions and beliefs about what should be expected from an intimate partner(ship)—whereby individuals with a more secure attachment orientation tend to have greater ease in being vulnerable and extending trust to close others, and those with a more insecure attachment orientation tend to be generally less trusting in their relationships (e.g., Mikulincer, 1998).

Whereas individual differences (e.g., attachment orientations) may represent a readiness to trust, an interdependence theoretical perspective suggests that experiences within a given relationship regulate trust’s development (Campbell & Stanton, 2019; Holmes & Rempel, 1989). Trust’s growth in relationships is theorized to be linked to the increase in interdependence between those involved—a view that guided Rempel et al.’s (1985) approach to the construct. Holmes and Rempel (1989) framed the development of trust in terms of uncertainty reduction whereby “… trust evolves out of successfully confronting increasing concerns about dependency as relationships develop” (p. 190). As partners become more interdependent, successfully reconciling issues raised by this increasing interdependency allows for trust to grow. Such issues take the form of, for example, finding oneself in positions of vulnerability or conflicting interests (Boon & Holmes, 1991; Simpson, 2007). In a similar vein, the development of trust is believed to be rooted in diagnostic situations—moments when the partner must navigate personal interests and those of the relationship (Kelley et al., 2001). Successfully confronting diagnostic situations can provide insight into the promise of the partner(ship) and, in turn, enrich trust. From this perspective, therefore, the trust people place in their romantic partners ought to grow over time as experiences accumulate and issues of interdependency are confronted (Simpson, 2007).

#### Paradox of trust

This account of trust however presents a paradox when compared with existing data: from the earliest through to the deepest stages of a romantic relationship, the amount of trust people place in their partner appears to remain appreciably high (e.g., Fletcher et al., 2000; Larzelere & Huston, 1980). Therefore, trust in a romantic partner might inherently exist at (or somewhere near) the maximum amount from the earliest stages of the relationship onward, potentially rendering accumulated experiences with the romantic partner an unnecessary precondition for developing trust over time.

#### Reimagining the development of trust

The pursuit of a deeper understanding of the nature of trust’s development is predicated on the manner in which *growth* is defined (Campbell et al., 2025). In the literal sense, the growth of trust can reflect a positive linear trend from relatively low to high amounts over time; in the figurative sense, the growth of trust can reflect a metamorphic construction of a person’s conceptualization of trust over time (Campbell et al., 2025). In our current research, we endeavoured to explore an account of trust that frames trust’s development as a process of construction in meaning, as opposed to addition in amount.

A defining feature of this view of trust is that it is responsive to important events and activities occurring within the confines of the relationship (Campbell & Stanton, 2019). That is, the major issues and themes in focus at the given period of a relationship’s development might give insight into the contents by which people construct their trust for their partner. Therefore, as an initial foray into reconciling the paradox of trust, we focused on investigating the (in)variability in the constructions of trust of individuals involved in newly-formed relationships versus those involved in long-term relationships.

### Description of research objectives and study

With our present research, we endeavoured to garner deeper insight into the nature of the construct of trust in romantic relationships. In Study 1, using data collected from a large sample of individuals involved in romantic relationships, we explored the form and essence of the construct of trust as put forward by Rempel et al. (1985). In Study 2, we confirmed our findings from Study 1, and then using our refined version of Rempel et al.’s (1985) assessment of trust, proceeded to capture the extent to which conceptualizations of trust across individuals involved in newly-formed relationships versus those involved in long-term relationships reflect different forms of the construct. We analyzed all our data using the *R* programming language (R Core Team, 2023). All our pre-registrations, data, analytic scripts, and supplementary materials are available on our Open Science Framework repository (https://osf.io/4km73/).

## Study 1

For Study 1, we evaluated Rempel et al.’s (1985) measurement framework for assessing trust as a way to gather deeper insights into the construct.

### Method

#### Participants and procedure

Study 1 included 494 individuals recruited from Prolific (https://www.prolific.co/). Eligible participants needed to be of legal age to consent, speak English fluently, and be in an ongoing romantic relationship for at least 2 years. Consenting participants were given a link to a Western Qualtrics survey. Upon completion, participants received a code to enter into the Prolific application to receive compensation (i.e., £2.00). We removed individuals who provided incorrect answers to any of the four attention check questions (*n =* 2). Participants had a *mean* age of 40.80 years (*Median* = 38, *SD =* 12.90, *Min =* 19, and *Max* = 79). Participants were based in either the United Kingdom (82.20%), Canada (11.90%), or the United States (4.25%). Most participants were non-student individuals (85.80%) and were employed in full-time (49.60%) or part-time work (18.60%). See Table 1 for a demographic breakdown of the Study 1 participants.

**Table 1.** Demographic breakdown of samples.

Demographic	Study 1 *N* = 494	Study 2 *N =* 847; *n*_Newly-formed_ = 387; *n*_Long-term_ = 460	
	*n* (%)	*n*_Newly-formed_ (%)	*n*_Long-term_ (%)
Sex			
Male	239 (48.40%)	165 (42.60%)	228 (49.60%)
Female	251 (50.80%)	217 (56.10%)	232 (50.40%)
Missing data	4 (.81%)	5 (1.29%)	0 (0%)
Gender			
Man	241 (48.80%)	155 (40.10%)	227 (49.30%)
Woman	249 (50.40%)	205 (53%)	228 (49.60%)
Transgender	0 (0%)	3 (.78%)	1 (.22%)
Agender	1 (.20%)	4 (1.03%)	0 (0%)
Another identity	2 (.41%)	19 (4.91%)	3 (.65%)
Prefer not to say	1 (.20%)	1 (.26%)	0 (0%)
Missing data	0 (0%)	0 (0%)	1 (.22%)
Sexual orientation			
Heterosexual	441 (89.3%)	256 (66.1%)	421 (91.50%)
Lesbian/Gay	9 (1.82%)	24 (6.20%)	16 (3.48%)
Bisexual	31 (6.28%)	71 (18.30%)	15 (3.26%)
Pansexual/Queer/Fluid	6 (1.21%)	27 (6.98%)	5 (1.09%)
Asexual	2 (.41%)	2 (.52%)	1 (.22%)
Another identity	2 (.41%)	2 (.52%)	1 (.22%)
Prefer not to say	2 (.41%)	5 (1.29%)	1 (.22%)
Missing data	1 (.20%)	0 (0%)	0 (0%)
Ethnicity			
African	2 (.41%)	8 (2.07%)	4 (.87%)
Black	5 (1.01%)	12 (3.10%)	8 (1.74%)
East Asian	6 (1.21%)	20 (5.17%)	13 (2.83%)
European	36 (7.29%)	28 (7.24%)	41 (8.91%)
Hispanic	9 (1.82%)	12 (3.10%)	7 (1.52%)
Indigenous	0 (0%)	0 (0%)	1 (.22%)
Middle Eastern	2 (.41%)	5 (1.29%)	3 (.65%)
Multi-ethnic	7 (1.42%)	13 (3.36%)	6 (1.30%)
South Asian	12 (2.43%)	9 (2.33%)	13 (2.83%)
Southeast Asian	10 (2.02%)	7 (1.81%)	10 (2.17%)
White	401 (81.20%)	270 (69.8%)	352 (76.50%)
Another identity	1 (.20%)	1 (.26%)	1 (.22%)
Prefer not to say	2 (.41%)	2 (.52%)	1 (.22%)
Missing data	1 (.20%)	0 (0%)	0 (0%)

*Note.* Age of Study 1 participants were, in years: *Mean* = 40.80, *Median =* 38, *SD =* 12.90, *Min =* 19, and *Max* = 79. Age of Study 2 *n*_Newly-formed_ participants were, in years: *Mean* = 27.40, *Median* = 25, *SD =* 8.14, *Min =* 18, and *Max* = 65. Age of Study 2 *n*_Long-term_ participants were, in years: *Mean* = 41.90, *Median* = 40, *SD =* 11, *Min =* 22, and *Max* = 79.

#### Predictability, dependability, and faith

We used Rempel et al.’s (1985) 26-item scale to capture participants’ trust in their romantic partner across the dimensions of predictability, dependability, and faith (see Supplementary Materials; https://osf.io/f32ds/). All items were evaluated on a 1 (strongly disagree) to 7 (strongly agree) scale and presented to participants in a randomized fashion.

#### Data analysis strategy

##### EFA

We used EFA, via the *psych* package (Revelle, 2024), to determine a psychometrically optimal and meaningful number of factors to represent Rempel et al.’s (1985) items. We employed maximum likelihood extraction with oblimin rotation to estimate factor solutions. EFA model selection was based on a combination of parallel analysis, nested model comparisons, model fit indices, and conceptual interpretability of factor loading patterns.

##### Construct multidimensionality due to fallible indicators

The construct multidimensionality present in assessments of psychological processes can emerge from: (1) the natural tendency of indicators to capture unintended processes; and/or (2) overarching latent constructs. These competing sources of construct multidimensionality, indeed, create ambiguity about whether the multidimensionality captured within a given assessment is best conceptualized as a consequence of the innate fallibility of the item contents at hand (e.g., presence of cross-loading indicators) or the hierarchical nature of the construct under study (e.g., presence of general factors) (Morin et al., 2016)—potentially undermining, in turn, the assessment’s construct validity. As Messick (1995) remarked, the major threats to the construct validity of assessments of psychological processes are *construct-irrelevant variance* and *construct underrepresentation*. Specifically, construct-irrelevant variance describes the degree to which the assessment and items therein tap into factors or processes that are adjacent or extraneous to the target construct (e.g., methodological artifacts), whereas construct underrepresentation describes the degree to which the assessment fails to capture the construct’s theoretical form and aspects (Messick, 1995; see Zumbo, 2023, for a review).

Given that Rempel et al.’s (1985) assessment is multidimensional yet designed to capture an overarching construct of trust, we used strategies described in Morin et al. (2016) to investigate the sources of construct multidimensionality present in our chosen exploratory measurement model and compilation of indicators. For our chosen model, we contrasted an Exploratory Structural Equation Model (ESEM) variant comprised of cross-loading indicators, versus a Confirmatory Factor Analysis (CFA) model variant comprised of fully discriminatory indicators. If the model’s fit and parameter estimates (i.e., factor correlations) for its CFA variant do not differ substantially from its corresponding ESEM variant, then the presence of construct multidimensionality due to the fallible nature of the model’s indicators can be deemed trivial. For further details regarding this approach, see Morin et al. (2016) as well as the Supplementary Materials.

The ESEM and CFA model variants, programmed via *lavaan* (Rosseel, 2012), were identified using the fixed-factor scale-setting and identification technique (Little, 2013), and fitted using robust maximum-likelihood estimation (MLR). Missing data were handled using full-information maximum-likelihood (FIML). Missingness accounted for approximately 0.13% of the analyzed data (see Supplementary Materials for further details). Though Little’s (1988) test indicated our missing data did not meet the MCAR (i.e., missing completely at random) assumption (*χ*^2^ (246) = 329, *p* < .05), FIML’s performance and handling of missing data (e.g., production of unbiased parameter and error estimates) has proven to be highly effective under missing data conditions driven by non-MCAR mechanisms—especially when the extent of the missingness is under 25% (Enders & Bandalos, 2001; Schlomer et al., 2010), as in our case (0.13% of missing data).

### Results

Observed means and standard deviations of trust, predictability, dependability, and faith for Study 1 are available in the Supplementary Materials.

#### EFA

The results of the parallel analysis suggested four factors were sufficient (see Supplementary Materials). Therefore, we evaluated solutions comprising of one-to-four factors (see Table 2). The evidence from nested model comparisons and model fit indices led us to further interpret and examine the four-factor solution (see Table 3) as a final candidate measurement model^1^.

**Table 2.** Model fit indexes and model comparisons for 1–4 exploratory factor analysis solutions (Study 1).

# Number of factors	RMSEA	TLI	BIC	∆BIC	*χ*^*2*^ (*df*)	*∆χ*^*2*^ (∆*df*)
1	.08	.84	−512.51		1342.05*** (299)	
2	.07	.89	−797.50	−284.99	902.00*** (274)	440.05*** (25)
3	.06	.93	−905.40	−107.90	645.24*** (250)	256.76*** (24)
4	.04	.96	−966.23	−60.84	441.74*** (227)	203.49*** (23)

*Note. N* = 494. RMSEA = root mean square error of approximation. TLI = Tucker–Lewis index. BIC = Bayesian Information Criterion. ***p < .001.

**Table 3.** Raw pattern matrix of candidate exploratory model (Study 1).

Item number and corresponding Rempel et al. (1985) subscale		Factor loadings				*h* ^*2*^	Retained?
		1	2	3	4		
Predictability							
*3.*	[R]	**.37**	**.38**	−.07	−.28	.20	
*5.*		.10	−.06	.11	**.66**	.67	Yes
*8.*	[R]	.03	**.45**	−.19	.06	.24	
*9.*	[R]	−.01	**.79**	.06	−.18	.77	
*12.*		.15	−.20	−.03	**.57**	.64	Yes
*17.*		.18	−.08	.16	**.56**	.68	Yes
*21.*	[R]	−.21	**.62**	.18	−.12	.59	
Dependability							
*2.*		**.67**	−.12	.09	.07	.72	Yes*
*4.*		.10	.02	**.43**	.25	.41	Yes
*10.*	[R]	−.19	**.45**	−.08	.18	.28	
*11.*		**.37**	.02	.10	.20	.32	Yes*
*19.*	[R]	−.01	**.66**	−.19	.05	.54	
*20.*		−.01	−.09	**.63**	.08	.50	Yes
*22.*		.33	−.06	**.47**	.14	.69	Yes
*25.*		.13	.03	**.53**	.20	.51	Yes
*26.*		**.49**	−.02	.13	−.06	.30	Yes*
Faith							
*1.*		.24	−.09	.22	.15	.32	
*6.*		**.74**	−.02	−.09	.01	.51	Yes
*7.*		**.60**	−.08	.24	.08	.74	Yes
*13.*		−.19	**.40**	−.32	.18	.42	
*14.*		**.71**	−.07	.11	−.01	.67	Yes
*15.*		**.38**	.02	**.38**	.08	.51	Yes
*16.*		**.86**	−.02	−.08	.03	.71	Yes
*18.*		**.78**	.01	.01	.09	.70	Yes
*23.*		−.18	.29	−.33	.08	.36	
*24.*		**.48**	−.05	.20	.22	.65	Yes

*Note. N* = 494. Item labels correspond to Rempel et al.’s (1985) 26-item trust scale. *h*^2^ = item communality. The parenthetical “R” denotes reverse-worded item. * = item that was originally proposed by Rempel et al. (1985) as an indicator of dependability. Bolded values indicate factor loadings that were considered substantial (i.e., |.35| or greater).

##### Close inspection and refinements

Loadings of |.35| or greater were deemed substantial. Recognizing that cross-loading indicators are not solely a threat to simple structure and can potentially contribute meaningfully to multidimensional construct spaces, selectively cross-loading indicators—substantially loading onto only a particular set of factors—were retained and permitted to be associated with the most conceptually compatible factor, whereas completely cross-loading indicators—(un)substantially loading onto all or no factors—were discarded (see Sakaluk & Fisher, 2019). Items 3 and 15 were identified as selectively cross-loading and retained, whereas items 1 and 23 were identified as completely cross-loading and discarded—leaving 24 indicators loading onto four factors. Compatible with Rempel et al.’s (1985) framework, one factor reflected indicators addressing trait attributions of the romantic partner’s character (dependability), whereas another factor reflected indicators addressing aspirational convictions in the romantic partner/relationship (faith). Of the remaining two factors, one factor reflected indicators addressing predictability whereas another factor reflected purely negatively worded indicators, potentially highlighting a demarcation of bona fide predictability content from general distrust content.

We decided to discard the indicators from the general distrust factor for two reasons. First, given that this family of indicators are all reverse-worded items their constellated factor might address methodological artifacts. Second, trust and distrust, though related, can be examined independently. Assuming trust and distrust form endpoints of a common continuum potentially goes beyond the scope of Rempel et al.’s (1985) proposed framework that was designed to capture the concept of trust.

A factor reflecting an abundance of negatively worded indicators was consistent with the findings of Rempel et al. (1985). Rempel et al. (1985) characterized their factor comprised of negatively worded items as a lack of predictability rather than the presence of predictability. However, they did not test a competitor 4-factor model as an underlying structure of their items and, thus, were unable to evaluate the degree to which bona fide predictability-related item content can be effectively demarcated from distrust-related item content (and/or methodological artifacts). Taken together, the final measurement structure suggested by our analyses contained 17 indicators capturing perceived partner predictability, perceived partner dependability, and faith (see Tables 2 and 4).

**Table 4.** ESEM and CFA model summaries (study 1).

Model	*df*	*χ* ^*2*^	Goodness of fit			Information criteria		Factor |*r*| range
			CFI	TLI	RMSEA	AIC	BIC	
ESEM variant	88	213.08	.96	.94	.05	23956.47	24301.08	.69 to .84
CFA model variant	116	268.42	.96	.95	.05	24015.45	24242.38	.79 to .83

*Note. N* = 494. *df* = Degrees of Freedom CFI = Comparative Fit Index. TLI = Tucker–Lewis index. RMSEA = root mean square error of approximation. AIC = Akaike Information Criterion. BIC = Bayesian Information Criterion.

#### Construct multidimensionality due to fallible indicators

Results from our investigations of sources of construct multidimensionality in Study 1 are summarized in Table 4 and the text below, but see Supplementary Materials for more details. In terms of goodness-of-fit indexes, both model variants—following standards outlined in Hu and Bentler (1999) (e.g., ≥.95 for the Comparative Fit Index [CFI] and Tucker-Lewis Index [TLI]; ≤.06 for the Root Mean Square Error of Approximation [RMSEA])—provided a tenable degree of fit to the data. In terms of information criteria, whereas the ESEM variant showed a slight improvement in model fit according to the Akaike Information Criterion (AIC), the CFA model variant showed a slight improvement in model fit according to the Bayesian Information Criterion (BIC). Model fit was therefore ambivalent for which variant ought to be favoured. Concerning parameter estimates, though the factor correlations differed across the ESEM (*r* = .69 to .84) and CFA variants (*r* = .79 to .83), the differences were not substantial, suggesting our chosen measurement structure may host a non-critical mass of indicators tapping multiple adjacent, non-target constructs (see Morin et al., 2016; as well as Supplementary Materials). Altogether, these results suggest the fallible nature of the indicators can be deemed a minimal source of the construct multidimensionality present in our chosen measurement structure.

### Discussion

In Study 1, we identified an interpretable and coherent compilation of items organized by underlying latent factors that appear to address constructs akin to Rempel et al.’s (1985) framework (see Table 5). One factor reflected the perceived predictableness of a romantic partner’s behavioural output; a second factor reflected trait attributions of the romantic partner’s dependability; and a third factor reflected faith in the romantic partner/relationship. Furthermore, the selected item compilation garnered support that precluded trust’s multidimensionality being sourced from the presence of items addressing multiple adjacent construct spaces. This finding may therefore hint at an overarching construct of trust as a potential source of trust’s multidimensionality—a possibility we address in Study 2.

**Table 5.** The final compilation of indicators.

Resulting factors and items	Designated subscale based on Rempel et al. (1985)
Predictability	
5.	Predictability
12.	Predictability
17.	Predictability
Dependability	
4.	Dependability
20.	Dependability
22.	Dependability
25.	Dependability
Faith	
2.	Dependability
6.	Faith
7.	Faith
11.	Dependability
14.	Faith
15.	Faith
16.	Faith
18.	Faith
24.	Faith
26.	Dependability

*Note.* Item numbering corresponds to that of Rempel et al.’s (1985) 26-item trust scale, whereas the categorization of items corresponds to conceptual interpretability of results derived from Study 1 (*N* = 494). See Supplementary Materials for item phrasings.

## Study 2

The accounts of trust theorists and lay notions assuming time as a necessary precondition for the development of trust seem to run paradoxical to the existing data (e.g., Fletcher et al., 2000; Larzelere & Huston, 1980). For Study 2, using our revised version of Rempel et al.’s (1985) assessment, we confirmed our findings from Study 1 and investigated (a) the appropriateness of an overarching general construct of trust, and (b) the extent to which the trust of individuals involved in new versus mature romantic relationships reflect different constructions.

### Bifactor modelling

Most researchers use a composite score of Rempel et al.’s (1985) measure to represent overall trust. In Study 2, we therefore investigated, via bifactor modelling (Holzinger & Swineford, 1937), the appropriateness of a psychometric structure of trust reflecting an overarching conceptualization (i.e., general factor) and specialized conceptualizations (i.e., specific factors). Bifactor modelling allows us to directly evaluate the nature of (a) variation across the full array of indicators caused by an overarching general construct of trust (i.e., general factor); and (b) variation across cluster sets of meaningfully grouped items caused by specific constructs concerning trust (i.e., predictability, dependability, and faith). See Supplementary Materials for a visual representation of the parameterization of a prototypical bifactor model.

### Invariance testing to investigate construction divergences

Measurement invariance techniques have profound utility for investigating (in)variability in the construction of psychological concepts and the nature of how such construction may unfold (see Sakaluk, 2019; Sakaluk et al., 2021). To examine the nature of trust’s construction as a function of romantic relationship maturation, we therefore contrasted, in a multigroup CFA framework, the trust of individuals involved in newly-formed relationships and those involved in long-term relationships, and demarcated construction divergences at the levels of configural, metric, and scalar invariance.

The test of configural invariance can capture (dis)similarities in trust’s form (i.e., number of factors and general pattern of loadings) across the modeled samples. The test of metric invariance—also often referred to as weak invariance or loading invariance—assesses whether the relations between the (observed) items and latent constructs (i.e., factor loadings) are equivalent across the modeled groups. Examinations into a measurement instrument’s metric invariance can, therefore, help to establish the extent to which the construct under study might carry (dis)similar meaning across the modeled groups (Sakaluk et al., 2021). The test of scalar invariance, also often referred to as strong invariance or intercept invariance, assesses whether the indicator intercepts (i.e., expected value of an item when its latent variable[s] is[are] at zero) are equivalent across the modeled groups (Sakaluk et al., 2021). Said differently, examinations into a measurement instrument’s scalar invariance can help to establish “whether an item has the same point of origin across different groups” (Chen, 2008, p. 1006).

### Method

#### Participants and procedure

Study 2 included 847 individuals—*n*_*Newly-formed*_ = 387 and *n*_*Long-term*_ = 460—recruited from Prolific (https://www.prolific.co/). Eligible participants needed to be of legal age to consent, speak English fluently, and be in a romantic relationship at the time of participation. The study was only made available to individuals reporting (via Prolific pre-screeners) being involved in an ongoing romantic relationship for 5 or more years (long-term relationships), or 6 months or less (newly-formed romantic relationships)^2^. Upon providing informed consent, participants were given a Western Qualtrics survey which included Rempel et al.’s (1985) 26-item trust scale as well as questionnaires assessing the nature of their relationship and demographic backgrounds. Upon completion, participants received a code to enter into the Prolific application to receive compensation (i.e., £3.00). We removed individuals who provided incorrect answers to any of the four attention check questions (*n =* 16). Participants had a *mean* age of 35.26 years (*Median* = 33, *SD =* 12.19, *Min =* 18, and *Max* = 79). Participants were based in either the United Kingdom (61.74%), the United States (32.59%) or Canada (5.67%). Most participants were non-student individuals (75.91%) and were employed in full-time (64.34%) or part-time work (21.25%). See Table 1 for a demographic breakdown of the Study 2 participants.

#### Predictability, dependability, and faith

Predictability, dependability, and faith were assessed using Rempel et al.’s (1985) 26-item Trust Scale—following the same administration method as Study 1.

#### Data analysis strategy

All analyses were performed using the *lavaan* (Rosseel, 2012) and *semTools* (Jorgensen et al., 2022) packages. All models were identified using the fixed-factor scale-setting and identification technique (Little, 2013) (see Supplementary Materials), and fitted using MLR; and missing data were handled using FIML. Missingness accounted for approximately 0.12% of the analyzed data (see Supplementary Materials for further details) and was—via Little’s (1988) test—demonstrated to be MCAR (*χ*^2^ [425] = 456, *p* = .145).

##### Investigation of sources of construct multidimensionality

###### Sources of construct multidimensionality due to fallible indicators

Sources of construct multidimensionality due to fallible indicators were investigated using the same data analysis strategy described in Study 1.

###### Sources of construct multidimensionality due to an overarching construct

Following a similar strategy described in Morin et al. (2016), we contrasted, via a CFA statistical framework, the correlated three-factor model variant^3^ and bifactor model variant on two levels of model evaluation: model fit and, more critically, the presence of a well-defined general factor. To the extent that the bifactor model variant shows improved model fit indices and the presence of a well-defined general factor, then the appropriateness of a bifactor structure and presence of construct multidimensionality due to an overarching construct would be supported. To appraise model fit of the three-factor and bifactor model variants, we placed a heavier premium on interpreting model fit indicators that included a correction for parsimony (i.e., TLI, RMSEA, AIC, and BIC) as the bifactor model is a heavily parameterized variant and subject to overfitting. To appraise the presence of a well-defined general factor, we took particular care in examining the extent to which the strength of the loadings onto the designated general factor could be demonstrated across a range of item contents.

#### Investigation of measurement model (in)variability

We tested the state of invariability of the final candidate measurement model between individuals in newly-formed relationships versus individuals in long-term relationships—examining measurement invariance at both the model level (i.e., configural, metric, and scalar invariance) and parameter level (i.e., invariability of particular items and model parameters) (Cheung & Lau, 2012; Cheung & Rensvold, 1999; Putnick & Bornstein, 2016). Measurement invariance was evaluated via fitting a series of increasingly constrained multi-group CFA models—incrementally constraining model parameters to equality across groups—and inspecting changes in model fit between each level of invariance constraints imposed. We began with a constraint of an identical quantity of latent factors and broad factor loading pattern (test of configural invariance), then proceeded to constrain the factor loading estimates to equality (test of metric invariance), and, lastly, proceeded to constrain the indicator variable intercepts to equality (test of scalar invariance).

Configural invariance was appraised using the permutation randomization method (see Jorgensen et al., 2018). Specifically, we used *I* = 1000 permutations via the *semTools* package (Jorgensen et al., 2022) to compute *p* values of statistical significance associated with ΔCFI and ΔRMSEA. Metric and scalar invariance were examined within a nested model comparison framework. Our adjudications of metric and scalar invariance were guided by the likelihood ratio test (i.e., Δ*χ*^*2*^), though—following the recommendations described in Khojasteh and Lo (2015)—we also computed ΔΓ and ΔCFI for descriptive purposes. Omnibus detections of metric and scalar non-invariance were further investigated by testing a series of models wherein the equality constraint of each indicator variable was released, and model fit was compared (i.e., via Δ*χ*^*2*^) against the corresponding fully constrained model (Cheung & Lau, 2012; Cheung & Rensvold, 1999; Putnick & Bornstein, 2016).

### Results

Observed means and standard deviations of trust, predictability, dependability, and faith for each sample are available in the Supplementary Materials.

#### Investigation of sources of construct multidimensionality

Summaries of our ESEM and CFA models are presented in Table 6.

**Table 6.** Model summaries for study 2.

Model	*df*	*χ* ^*2*^	Goodness-of-fit			Information criteria		Factor |*r*| range
			CFI	TLI	RMSEA	AIC	BIC	
Correlated three-factor structure								
ESEM variant	88	270.01	.97	.95	.05	42103.58	42492.40	.73 to .82
CFA model variant	116	337.41	.96	.96	.05	42188.10	42444.15	.80 to .87
*Bifactor structure*	102	213.93	.98	.97	.04	42023.13	42345.57	

*Note. N* = 847. *df* = Degrees of Freedom. CFI = Comparative Fit Index. TLI = Tucker–Lewis index. RMSEA = root mean square error of approximation. AIC = Akaike Information Criterion. BIC = Bayesian Information Criterion.

##### Construct multidimensionality due to fallible indicators

In terms of goodness-of-fit, each of the correlated three-factor ESEM (i.e., containing estimated cross-loadings) and CFA model (i.e., fully discriminatory indicators) variants provided a tenable degree of fit to the data—according to Hu and Bentler (1999) cut-offs. However, whereas the ESEM variant was suggested to provide an improvement in model fit according to AIC, the CFA model variant was suggested to provide an improvement in model fit according to BIC. Thus, the model fit was ambivalent for which model variant ought to be favoured and, in turn, preliminarily precluded the presence of items inadvertently addressing multiple adjacent non-target construct spaces as a potential primary source of construct multidimensionality. Furthermore, although the factor correlations among the ESEM (|*r*| = .73 to .82) and CFA model (|*r*| = .80 to .87) variants differed, the differences were, descriptively, not substantial.

The pattern of findings suggested that, though the model fit to the data and latent factor correlations of the CFA model variant differed from those of the corresponding ESEM variant, the extent of the differences was, descriptively, not vast—thereby replicating and confirming the chosen exploratory model of Study 1. Indeed, of the construct psychometric multidimensionality represented in the candidate measurement structure under consideration (i.e., correlated three-factor model), the presence of items inadvertently addressing multiple adjacent nontarget construct spaces could be deemed a minimal source—which, in turn, spoke to the tenability of retaining the CFA model variant (i.e., modelling fully discriminatory indicator variables) for further testing. Therefore, using the CFA model variant, we proceeded to test the presence of an overarching construct of trust as an alternate source of construct multidimensionality.

##### Construct multidimensionality due to an overarching construct

Although both the correlated three-factor model and the bifactor model variant provided a tenable degree of fit to the data, the bifactor model showcased superior fit to the data according to the indicators of model fit that includes a correction for parsimony and penalty for over-parameterization. The full array of loadings and intercepts of the bifactor model are outlined in Table 7.

**Table 7.** Base (Generalized across samples) bifactor model (Study 2).

Items and specific factors	*λ*_G_	*λ*_S_	*τ*
Predictability			
*5.*	.57***	.44***	4.17
*12.*	.61***	.53***	3.73
*17.*	.73***	.42***	3.74
Dependability			
*4.*	.62***	.47***	3.26
*20.*	.59***	.44***	4.16
*22.*	.76***	.26***	3.69
*25.*	.62***	.36***	3.96
Faith			
*2.*	.84***	−.27*	5.11
*6.*	.70***	.30*	3.18
*7.*	.86***	−.08	4.58
*11.*	.58***	−.09	4.06
*14.*	.83***	−.30***	3.57
*15.*	.69***	.12*	4.19
*16.*	.80***	.10	3.00
*18.*	.83***	.05	3.68
*24.*	.76***	.05	4.75
*26.*	.57***	.16***	4.50

*Note. N* = 847. *p < .05. **p < .01. ***p < .001. *λ*_G_ = Standardized loading onto general factor. *λ*_S_ = Standardized loading onto specific factor. *τ* = indicator intercept. Item numbering corresponds to that of Rempel et al.’s (1985) 26-item trust scale. See Supplementary Materials for item phrasings and further details of the model’s parameters.

The bifactor model showcased that the overarching general factor of trust was well-defined by the presence of strong, significant target loadings across the full array of items (|λ| = .57 to .86, *Median* = .70). More specifically, however, the faith items (|λ| = .57 to .86, *Median* = .78) demonstrated elevated target loadings on the overarching general factor in comparison to the predictability (|λ| = .57 to .73, *Median* = .61) and dependability items (|λ| = .59 to .76, *Median* = .62). Over and above the overarching general factor of trust, the specific factors of predictability (|λ| = .42 to .53, *Median* = .44) and dependability (|λ| = .26 to .47, *Median* = .40) were also well-defined by virtue of appreciable target loadings. This suggests that the predictability and dependability items not only reflect the general content substance shared across the full array of items, but also tap into relevant specificity and address additional information beyond the general factor. The specific factor of faith, in contrast, was showcased to be defined by virtue of a compilation of appreciable as well as nonsignificant target loadings (|λ| = .05 to .30, *Median* = .09). This suggests that some faith items seem to reflect both the generality of the content substance shared across the full array of items as well as an appreciable degree of relevant specificity beyond the general factor, whereas the remaining faith items seem only to reflect the former.

Taken together, these findings speak to the appropriateness of an overarching concept and bifactor structure underlying responses to the compilation of item contents. We therefore chose the bifactor model variant as the final measurement model of trust and proceeded to our tests of invariance.

#### (in)Variability of the measurement model

##### Model-level (in)Variability

Summaries of our multigroup CFA models are presented in Table 6, whereas summaries of our model-level invariance tests are presented in Table 8. We submitted the chosen measurement model of trust (i.e., the bifactor model) to a suite of invariance tests on individuals involved in newly-formed romantic relationships versus individuals involved in long-term romantic relationships (*n*_*Newly-formed*_ = 387 vs. *n*_*Long-term*_ = 460)^4^. The loadings and intercepts of the configural invariance model are outlined in Table 9. This model had acceptable fit across the two independent samples (CFI = .98) according to Hu and Bentler’s (1999) cut-offs. More importantly, permutation tests using both CFI and RMSEA supported configural invariance (ΔCFI = .97, *p* = .441; ΔRMSEA = .053, *p* = .421). In comparison to the configural invariance model, the metric invariance model was found to fit significantly worse (Δ*χ*^*2*^[30] = 67.35, *p* < .001). Finally, in comparison to the metric invariance model, the scalar invariance model was found to fit significantly worse (Δ*χ*^*2*^[13] = 84.75, *p* < .001). Across the two modelled samples, the pattern of results suggests that the theoretical structure of the model was invariant configurally, yet the model seemed to be demonstrating non-invariability at the level of the loading and intercept parameters. Furthermore, a sensitivity analysis indicated that the imbalance between the modelled sample sizes was unlikely to have any practical impact on the outcomes of our model-level invariance tests (see Footnote 4).

**Table 8.** Model-level invariance (Study 2).

	*df*	Goodness-of-fit				Information criteria		PRT (*I* = 1000 permutations)		Model comparison				
										LRT			ΔΓ	∆CFI
		*χ* ^*2*^	CFI	TLI	RMSEA	AIC	BIC	∆CFI	∆RMSEA	***Δ*** *χ* ^*2*^	***Δ*** *df*	*p*		
Configural	204	334.03	.98	.97	.04	41908.18	42553.06	.97, *p* = .441	.053, *p* = .421					
Metric	234	401.53	.97	.97	.04	41938.30	42440.92			67.35	30	<.001	−.008	−.006
Scalar	247	466.03	.96	.96	.05	41986.91	42427.89			84.75	13	<.001	−.008	−.007

*Note. N* = 847. *n*_Newly-formed_ = 387. *n*_Long-term_ = 460. *df* = Degrees of Freedom. CFI = Comparative Fit Index. TLI = Tucker–Lewis index. RMSEA = root mean square error of approximation. AIC = Akaike Information Criterion. BIC = Bayesian Information Criterion. PRT = Permutation Randomization Test. LRT = Likelihood Ratio Test. Γ = Gamma.

**Table 9.** Loadings & intercepts of configural invariance bifactor model (Study 2).

Items and specific factors	*n*_*Newly-formed*_ *=* 387			*n*_*Long-term*_ *=* 460		
	*λ*_G_	*λ*_S_	*τ*	*λ*_G_	*λ*_S_	*τ*
Predictability						
*5.*	.57***	.41***	4.29	.58***	.45***	4.09
*12.*	.59***	.58***	4.24	.62***	.51***	3.42
*17.*	.75***	.40***	3.85	.72***	.41***	3.66
Dependability						
*4.*	.64***	.40***	3.47	.62***	.49***	3.11
*20.*	.58***	.42***	4.54	.59***	.47***	3.89
*22.*	.76***	.25***	3.76	.77***	.28***	3.64
*25.*	.61***	.25***	3.95	.63***	.43***	3.96
Faith						
*2.*	.78***	.34*	5.91	.89***	−.19	4.64
*6.*	.65***	−.13	3.34	.71***	.47***	3.06
*7.*	.83***	.05	5.04	.90***	−.09	4.28
*11.*	.52***	.14	4.22	.62***	−.05	3.94
*14.*	.77***	.43***	4.30	.86***	−.19*	3.17
*15.*	.68***	.06	4.45	.67***	.26***	4.00
*16.*	.72***	−.11	2.92	.84***	.14	3.07
*18.*	.75***	.05	3.96	.88***	.16	3.48
*24.*	.74***	−.05	4.89	.77***	.04	4.66
*26.*	.55***	−.14*	4.56	.59***	.15*	4.46

*Note. N* = 847. *p < .05. **p < .01. ***p < .001. *λ*_G_ = Standardized loading onto general factor. *λ*_S_ = Standardized loading onto specific factor. *τ* = indicator intercept. Item numbering corresponds to that of Rempel et al.’s (1985) 26-item trust scale. See Supplementary Materials for item phrasings and further details of the model’s parameters.

##### Parameter-level (in)Variability

Due to the number of identified sources of non-invariance, we outline the patterns of loading and intercept non-invariability that we believe to be most relevant. We sought to establish partial invariance by relaxing the minimum number of equality constraints necessary to achieve a fit comparable to the baseline model (i.e., a nonsignificant Δ*χ*^*2*^ test) (see Jorgensen et al., 2018). Parameters producing the largest resulting decreases in *χ*^*2*^ were prioritized for relaxation (see Table 10 as well as the Supplementary Materials).

**Table 10.** Parameter-level invariance test results (Study 2).

Items and specific factors	G factor metric invariance		Scalar invariance	
	***Δ*** *χ* ^*2*^	*p*	***Δ*** *χ* ^*2*^	*p*
Predictability				
*5.*	.00	.96	1.32	.25
*12.*	.17	.68	6.77	.01
*17.*	4.26	.04	2.51	.11
Dependability				
*4.*	2.04	.15	2.41	.12
*20.*	.01	.93	7.54	.01
*22.*	.11	.75	1.41	.23
*25.*	.40	.53	4.45	.03
Faith				
*2.*	3.03	.08	15.57	<.001
*6.*	.02	.88	.19	.66
*7.*	2.90	.09	34.75	<.001
*11.*	.00	.97	.14	.70
*14.*	.02	.89	.26	.61
*15.*	3.99	.05	2.54	.11
*16.*	9.34	<.01	5.36	.02
*18.*	33.22	<.001	30.71	<.001
*24.*	1.12	.29	.71	.40
*26.*	1.87	.17	8.06	<.001

*Note. N* = 847. *n*_Newly-formed_ = 387 involved in newly-formed romantic relationships. *n*_Long-term_ = 460 involved in long-term romantic relationships. Item numbering corresponds to that of Rempel et al.’s (1985) 26-item trust scale. See the Supplementary Materials for item phrasings.

###### Partial metric invariance model

We fitted a candidate partial metric invariance model by relaxing the general factor loadings from items 16 and 18 as well as relaxing the faith factor loadings from items 14 and 15. This partial metric invariance model did not fit significantly worse than the configural invariance model (Δ*χ*^*2*^[26] = 33.73, *p* = .142) and was, therefore, retained. Items 16 and 18 comprised loadings onto the general factor that were revealed to be stronger for individuals involved in long-term relationships than for individuals involved in newly-formed relationships. Such non-invariability might suggest the contents expressed in items 16 (*“I can rely on my partner to react in a positive way when I expose my weaknesses to him/her”*) and 18 (“*When I share my problems with my partner, I know he/she will respond in a loving way even before I say anything*”) resonate more with conceptualizations of trust within relationships that carry denser shared history, while taking on relatively lesser importance in how individuals in early-stage relationships may conceptualize the construct.

Among the relaxed faith factor loadings, the loading for item 14 was revealed to be stronger for individuals in newly-formed relationships, whereas the loading for item 15 was revealed to be stronger for those in long-term relationships. Despite these group differences, neither sample supported the emergence of a well-defined residualized factor from the modeled compilation of faith-based indicators (see Table 9). Thus, the parameter-level non-invariance identified within the faith factor should be interpreted with caution, as poorly defined specific factors can lack substantive meaning.

##### Partial scalar invariance model

The scalar invariance model constrained all intercepts of the retained partial metric invariance model to equality across individuals in newly-formed relationships versus those in long-term relationships. The scalar invariance model fit significantly worse than the partial metric invariance model (Δ*χ*^*2*^[13] = 79.44, *p* < .001), indicating the presence of non-invariant intercepts. Following the diagnosis of intercept parameters responsible for the omnibus detection of scalar non-invariance (see Table 10), we fitted a partial scalar invariance model by freeing the intercepts of items 2, 7, 12, 18, 20, and 26. This partial scalar invariance model did not fit significantly worse than the partial metric invariance model (Δ*χ*^*2*^[7] = 11.18, *p* = .131) and was, therefore, retained (i.e., no further modifications were examined). Those in newly-formed relationships exhibited higher indicator intercepts on all freed items compared to those in long-term relationships. Among the six items freed in the partial scalar invariance model, four comprised faith-based content (e.g., *“I am willing to let my partner make decisions for me”* [Item 26]), while the remaining two comprised predictability-based (*“My partner behaves in a very consistent manner”*) and dependability-based (*“I am certain that my partner would not cheat on me, even if the opportunity arose and there was no chance that he/she would get caught”*) content.

### Discussion

In Study 2, we found support for the appropriateness of a general construct underlying responses to our revised version of Rempel et al.’s (1985) assessment. Predictability and dependability item contents appeared to address the general construct as well as relevant specificity associated with their respective specialized constructs, whereas most faith items appeared to only address the general construct. Importantly, the model- and parameter-level tests of invariance garnered evidence supporting the notion that individuals in newly-formed relationships and individuals in long-term relationships may conceptualize trust for the romantic partner in non-equivalent ways. Although there was evidence for configural invariance, the evidence for metric and scalar non-invariability across individuals in newly-formed versus long-term relationships may reflect interpretive differences of the item contents and, in turn, potential divergences in the construction of trust. We discuss the implications of these results in the General Discussion.

## General discussion

A goal of this research was to enhance our understanding of the nature of trust in romantic relationships by assessing the measurement as well as invariance of the construct between large samples of individuals in newly-formed compared to long-term romantic relationships. Some important implications of our research and results are that (a) we replicated the factor structure of trust as conceptualized and assessed by Rempel et al. (1985) but with the important caveat that the predictability factor is better represented by positively-worded items than the negatively-worded items suggested by the original analyses; (b) the development of trust does not appear to begin at relatively lower levels in newly-formed relationships compared to relationships that have existed for a longer time; and, (c) according to the measurement invariance analyses there (i) is a great deal of consistency in the construct of trust between newly-formed and long-term relationships, but also (ii) some important differences that potentially suggest how trust might be conceptualized and/or measured differently over time. In the following sections, we discuss these implications for the field’s understanding of the nature of trust, including additional future research needed to test the possibilities suggested by our findings.

### Measurement of trust

Rempel et al.’s (1985) theory-based measure of trust is immensely popular, largely owing to its reputation for offering a greater depth of insight into the theoretical entity compared to other comparable measurement instruments (e.g., Johnson-George & Swap, 1982; Larzelere & Huston, 1980). However, the quality of the data and methods employed in Rempel et al. (1985) fall short of modern standards, and research building on their foundation with further (and ongoing) investigation has been absent. With large samples of individuals involved in romantic relationships, our psychometric investigation yielded a resulting measurement instrument and model that broadly dovetails with Rempel et al.’s (1985) conceptualization, as well as garnered psychometrically informed insights on how to better model trust’s elements.

In line with Rempel et al. (1985), our results pointed to a multidimensional, tripartite conceptualization of trust reflecting a demarcation of predictability-, dependability-, and faith-based item contents. Our resulting measurement instrument, however, assesses these constructs through a compilation of items slightly different from those selected by Rempel et al. (1985). Nevertheless, such differences in the item compilations proved necessary for reclaiming a sound measurement structure that upholds the hierarchical nature of trust (i.e., an overarching, general construct of trust) and, in turn, an overall composite score of trust (see Morin et al., 2016; Sakaluk & Fisher, 2019)—representing an improvement that should benefit future research using this modified scale.

Furthermore, representing trust’s multidimensionality and hierarchical organization under a bifactor model (i.e., a coexistence of a general construct and specific subconstructs) proved fruitful in terms of empirical adequacy and for garnering insight into the properties of the individual test items and substance of the constructs being assessed by those items. We found support for a general construct of trust that was defined most strongly via faith-based items. Additionally, our findings suggested that whereas predictability and dependability-based items can address meaningful residual specificity (i.e., information that is not conveyed by the general construct), the majority of faith-based items offered little meaningful residual specificity and instead reflected pure indicators of the general construct. An important step for future investigations on the measurement front of trust research would be to uncover item contents that can effectively address not only trust but also substantive faith specificity. Future forays into the content substance of the faith that romantically-attached individuals hold for their partner may particularly benefit from the use of qualitative methods (e.g., interviews, text analysis, etc.) and item response theory techniques (e.g., De Ayala, 2009).

### Development of trust

It is generally assumed that trust grows over time as experiences accumulate. Such accounts may imply that the trust felt toward a partner should start at a relatively low amount from the initial periods of a relationship and then grow through the gathering of experiences with the partner. Our results, however, help to provide psychometric context and further clarity for prior descriptive data documenting people’s trust being at a heightened state regardless of whether they are involved in a new or established relationship.

Our results suggest that when in romantic relationships, people are trusting of their partners as a default, with feelings of trust not requiring time and experiences with a partner to develop from lower to higher levels. Several possible implications stem from this finding. For instance, only for certain individuals may trust in a romantic partner develop (e.g., grow and/or decline) because of relationship-specific experiences. The interplay of attachment orientation and trust development in particular might offer a promising avenue of inquiry (see Campbell & Stanton, 2019). Furthermore, although people may report high levels of trust for their partner across all stages of the relationship, the nature of how they express their trust to their partner behaviourally may vary in important ways. Future investigations into the nature of trust’s development may benefit from the inclusion of measurement instruments that target the behaviours of trust (e.g., Kleinert et al., 2020).

### Measurement invariance and construction of trust

#### Construction convergences

Those in newly-formed relationships and those in long-term relationships were revealed to possess a broadly invariant structure of trust. The trust (i.e., general factor) of both those involved in newly-formed relationships and those involved in long-term relationships was most strongly defined by faith items rather than predictability or dependability items. Yet both samples seemed to possess strongly defined specific constructs of only predictability and dependability, while faith remained a weakly defined specific construct. Such patterns of sameness may speak to the (un)importance of the roles faith and evidence play in people’s trust throughout the relationship’s timeline.

Regardless of the stage of relationship development, faith may reflect the most important contents by which romantically-attached individuals construct their conceptualization of trust, whereas evidence may take on a more ancillary function. For instance, though evidence may not serve as a necessary precondition for the development of high trust, evidence may instead serve to calibrate trust (to a fitting amount). If trusting romantic partners at a relatively high level is the default, then evidence should only assume an appreciable role in trust’s development when perceived as contradictory to such certainty. As individuals confront diagnostic situations and issues of maturing interdependency, past experiences concerning trust may be susceptible to interpretation and/or dismissal (Fletcher & Kerr, 2010; Luchies et al., 2013; Norona et al., 2017). While trust should suffer a breakage at the hands of a major betrayal (e.g., discovering a partner’s infidelity), a breakage may also result from a series of minor negative experiences that are trivial in isolation but coalesce over time into a bigger problem that can no longer be overlooked. Future research should endeavour to tease apart the roles of faith and evidence in trust’s development and junctures by which evidence may precipitate a breakage in one’s trust.

#### Construction divergences

Some of the non-invariance that emerged highlight potential areas of the trust construct that may differ as a function of relationship maturation. For the majority of the items responsible for scalar non-invariance, individuals in newly-formed relationships had substantially higher indicator intercepts compared to those in long-term relationships. Specifically, when latent factors are at baseline (i.e., zero), the expected values of item responses were higher for individuals in newly-formed relationships across a broader range of item contents than for those in long-term relationships. This non-invariability may speak to the audaciousness of trust in new relationships. Any uncertainties of the initial periods of the partnership are susceptible to being dampened by early romantic feelings and thus left unchallenged (Boon & Holmes, 1991; Fisher, 1998; Hatfield et al., 1988). Having such confidence in the partner and relationship’s future may in part function to provide opportunity for the relationship to survive the uncertainty inherent in the early stages of development.

In contrast to those in newly-formed relationships, statements of faith like “I can rely on my partner to react in a positive way when I expose my weaknesses to him/her” and “When I share my problems with my partner, I know he/she will respond in a loving way even before I say anything” were revealed to define the general factor substantially more for those involved in long-term relationships. Certainty in being met with positivity and love from the partner within positions of vulnerability might be especially central to the meaning of trust for those involved in long-term relationships, while taking relatively lesser psychological significance in the trust of those involved in newly-formed relationships. Whereas uncertainty concerning a newfound partner can be overshadowed by early romantic love, people may later become more aware of the perils of disappointment and rejection as the relationship deepens. Thus, as romantically-attached individuals progress deeper into the relationship, confidence in the partner’s goodwill and responsiveness in the context of vulnerability may gain increasingly more importance and become a prominent theme in people’s trust.

### Recommendations and precautions

Based on the results of our investigations we therefore offer the following recommendations and precautions for future work involving trust within romantic relationships. Given that researchers typically use the Rempel et al. (1985) scale to calculate an overall score of trust for their analyses, we strongly recommend using our modified compilation of Rempel et al.’s (1985) item contents (see Table 5). The creation and use of an overall composite score presume the presence of a generalized factor underlying the diversity of items involved—a measurement structure in need of formal support (Morin et al., 2016; Sakaluk & Fisher, 2019). Via our psychometric efforts, we identified a compilation of items that demonstrated a coherent generalized factor, offering users an assessment that can reliably yield an unambiguous overall score of trust.

To the extent researchers are interested in assessing the specific theorized content domains of trust independently, we also recommend using our modified item compilation—namely the subsets of item contents contained therein (see Table 5). Our identified subsets of predictability- and dependability-based item contents have a capacity for tapping into meaningful residual specificity beyond generalized trust, providing downstream users with reliable (short) assessments of perceptions of a partner’s predictability and dependability. However, caution should be exercised in using our resulting subset of faith-based items to create a stand-alone index of faith. Unlike predictability and dependability, our findings indicated that the resulting subset of faith-based item contents broadly reflects the generalized construct and addresses little-to-no meaningful faith specificity. We recommend researchers perform a bifactor analysis to examine the extent to which their chosen compilation of Rempel et al.’s (1985) items can, under the unique conditions of their data, reflect a coherent generalized construct (i.e., trust) as well as reflect meaningful residualized (sub)constructs (e.g., predictability, dependability, and faith). Doing so can garner useful insights into the appropriateness of a generalized factor of trust as an underlying structure of the user’s data and in turn offer them demonstrable support for the generation and use of composite scores for overall trust as well as for predictability, dependability, and/or faith.

Furthermore, although tests of measurement invariance showcased that our modified compilation of Rempel et al.’s (1985) items broadly performed in a similar manner across individuals involved in different stages of relationship maturation (i.e., newly-formed vs. long-term relationships), some items were found to perform differently (i.e., metric and scalar non-invariance) across these groups. We therefore recommend researchers continue to examine the nature of the measurement invariance of Rempel et al.’s (1985) items across individuals involved in various relationship stages, as well as across other grouping variables that may drive noninvariant assessments (and conceptualizations) of trust (e.g., culture, sexual orientation, relationship structure, etc.). Such forays into the psychometric (non)invariability of Rempel et al.’s (1985) items—as well as the item contents of other instantiations of trust—will prove useful not only in uncovering a generalizable assessment of trust, but also for garnering deeper psychological insights into the (dis)similarities in people’s conceptualization of the intra-psychic entity (see Sakaluk, 2019; Sakaluk et al., 2021).

### Limitations and final considerations

The findings from our research should be interpreted with some limitations in mind. Our studies were conducted with samples comprised predominantly of white, heterosexual individuals—potentially limiting the generalizability of our findings. Information on specific socio-demographic variables—such as disability status, household size, and income—was not collected in this research. Furthermore, our research contrasted trust across two samples of individuals involved in relationships of vastly different durations, yet the nature of trust’s development in relationships may require intensive longitudinal designs to capture fully.

Overall, the research we presented derived from a process of evaluating core assumptions of Rempel et al.’s (1985) theory and measurement of trust and comparing them to existing evidence. Whereas Rempel et al.’s (1985) trust scale has been employed heavily in subsequent research, no research has systematically evaluated the measurement model of the instrument nor the theoretical speculations regarding the growth of trust in relationships. Our research attempted to redress these important gaps—providing consistent and strong support for a modified version of Rempel et al.’s (1985) original trust scale as well as shining some light on the nature of trust in the early and later stages of relationship development. We hope our research efforts can spur a research direction toward treating the concept of trust in romantic relationships as an important and interesting analytic outcome and topic of study in its own right.

## Notes

1. The three-factor EFA solution, although evidenced to possess only somewhat reasonable fit to the data, was also closely examined in tandem with the four-factor EFA solution. See Supplementary Materials.

2. Our chosen criteria for distinguishing newly-formed relationships—romantically involved for 6 months or less—versus long-term relationships—romantically involved for 5 years or more—were loosely based on theories and stage models of relationship development (e.g., Altman & Taylor, 1973; Knapp, 1978; Murstein, 1970). Theoretical models of relationship development typically center on the *qualitative experiences* separating earlier versus later stages of relationship maturation rather than the *duration*, given that relationships can progress at drastically different speeds. That is, time is thought not necessarily to be the primary mechanism of relationship development but instead, a proxy that can reflect and provide insight into the growth of a relationship. Our chosen criteria were therefore designed as pragmatic guidelines for recruiting participants involved in different stages of their relationship, rather than to claim specific turning points or developmental milestones of a romantic relationship.

3. A three-factor model’s first-order correlated-factor variant and second-order factor variant (i.e., the same three first-order factors defining a single second-order factor) are mathematically equivalent (Morin et al., 2016). Therefore, of the two, we chose only to specify and interpret the correlated three-factor model variant.

4. Given the unbalanced sample sizes in the modeled groups of individuals involved in newly-formed relationships (*n* = 387) versus individuals involved in long-term relationships (*n* = 460), we performed a sensitivity analysis via implementing an approach similar to that described in Yoon and Lai (2018). Over 100 iterations, we examined measurement invariance (i.e., configural, metric, and scalar invariance) between the (original) sample of *n* = 387 individuals involved in newly-formed relationships and a randomly drawn subsample of *n* = 387 respondents from the (original) sample of individuals involved in long-term relationships. Our tests of measurement invariance across all 100 iterations echoed the findings from the original study. Supplementary files—full analysis strategy, scripts, and tabularized and visualized output—are available in our Open Science Framework repository.
